# Carotid plaque vulnerability and circle of Willis anatomy predict ipsilateral brain infarcts and long-term mortality in carotid endarterectomy patients

**DOI:** 10.1007/s11357-026-02327-3

**Published:** 2026-05-23

**Authors:** Andrea Varga, Milán Vecsey-Nagy, Csongor Péter, Andrea Lehoczki, Péter Banga, Balázs Lengyel, Sarolta Borzsák, Gyopár Bálint, Zoltán Ungvári, Péter Sótonyi

**Affiliations:** 1 Department of Diagnostic Radiology, Heart and Vascular Center, Semmelweis University, 18. Határőr Street, Budapest, Hungary; 2 Department of Interventional Radiology, Heart and Vascular Center, Semmelweis University, Budapest, Hungary; 3 Department of Radiology and Radiological Science, Medical University of South Carolina, Charleston, SC USA; 4 Institute of Preventive Medicine and Public Health, Semmelweis University, Budapest, Hungary; 5 Fodor Center for Prevention and Healthy Aging, Semmelweis University, Budapest, Hungary; 6 Department of Vascular and Endovascular Surgery, Heart and Vascular Center, Semmelweis University, Budapest, Hungary; 7 Vascular Cognitive Impairment, Neurodegeneration and Healthy Brain Aging Program, Department of Neurosurgery, University of Oklahoma Health Sciences Center, Oklahoma City, OK USA; 8 Oklahoma Center for Geroscience and Healthy Brain Aging, University of Oklahoma Health Sciences Center, Oklahoma City, OK USA; 9 Stephenson Cancer Center, University of Oklahoma, Oklahoma City, OK USA; 10 Department of Health Promotion Sciences, College of Public Health, University of Oklahoma Health Sciences Center, Oklahoma City, OK USA; 11 Doctoral College/Department of Public Health, International Training Program in Geroscience, Semmelweis University, Budapest, Hungary

**Keywords:** Vascular aging, Stroke, Atherosclerosis, Carotid endarterectomy, Carotid artery stenosis, CT angiography, Carotid plaque vulnerability, Plaque ulceration, Watershed infarcts, Territorial infarcts, Circle of Willis, Cerebral ischaemia, Brain infarcts, Collateral circulation, Mortality

## Abstract

Cerebral infarcts in carotid artery disease arise from a complex interplay between plaque vulnerability, extracranial vascular burden, intracranial collateral capacity. Although each of these factors has been studied individually, their combined impact on infarct presence, infarct pattern, and long-term outcomes in real-world carotid endarterectomy (CEA) populations remains insufficiently understood. This study aimed to determine how carotid plaque morphology, bilateral carotid stenosis severity, circle of Willis (CW) anatomy jointly influence ipsilateral brain infarcts and overall mortality. We retrospectively analyzed 1,304 CEA patients using standardized CT and CT angiography to assess plaque features, maximal plaque thickness, carotid stenosis burden (carotid score), detailed CW configuration. Ipsilateral cerebral infarcts were classified as territorial or watershed. Logistic regression was used to determine predictors of infarcts and infarct pattern, Cox proportional-hazards models assessed long-term mortality. Reproducibility of plaque composition and thickness assessment was evaluated in 100 patients using Cohen’s kappa and intraclass correlation coefficient. Ipsilateral infarcts were present in 36.7%. Independent predictors included symptomatic status, greater plaque thickness, exulcerated plaques, more advanced bilateral carotid disease (lower carotid score), severely compromised CW. Lower carotid scores predicted watershed infarcts, supporting a hemodynamic mechanism; exulcerated plaques predicted territorial infarcts, consistent with distal embolization. Long-term mortality was independently associated with age, diabetes mellitus, chronic obstructive pulmonary disease, severe chronic kidney disease, the presence of ipsilateral infarcts, type-1 CW anatomy, and calcified plaques. In conclusion, plaque vulnerability, extracranial vascular burden, CW integrity provide complementary, clinically meaningful information on the development and pattern of brain infarcts and on long-term survival after CEA. These findings underscore the importance of comprehensive cerebrovascular imaging for risk stratification and individualized management of carotid artery disease.

**Graphical Abstract:**

**Figure:**
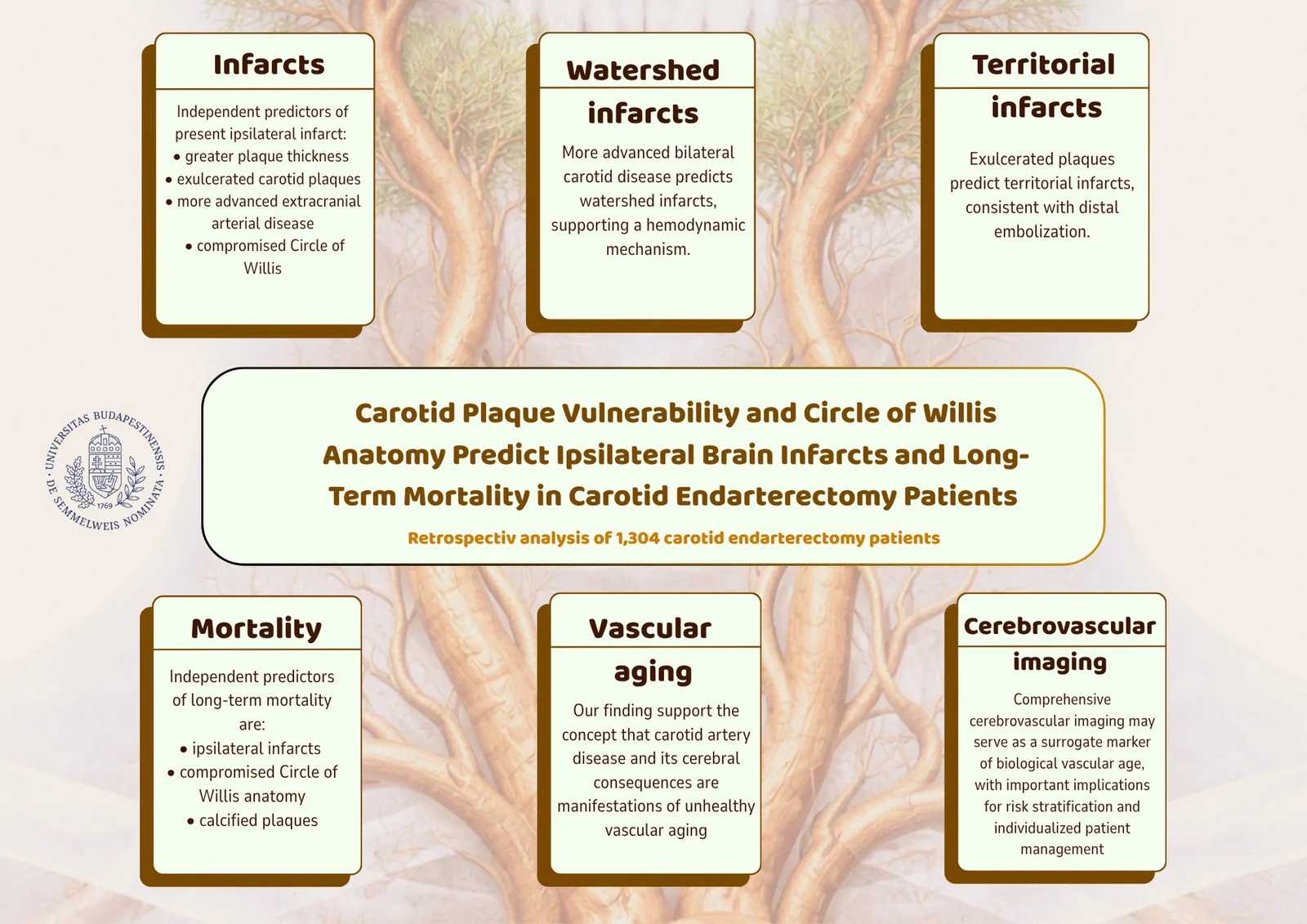

## Introduction

Carotid artery stenosis has long been recognized as a major determinant of ischaemic stroke, and stenosis grade traditionally serves as the primary criterion for risk stratification and indications for carotid revascularization [1]. However, accumulating evidence shows that stenosis severity alone does not fully account for the variability in clinical presentation or outcomes among patients with comparable degrees of luminal narrowing. Certain plaque characteristics—such as lipid-rich necrotic core, intraplaque haemorrhage, and surface ulceration—render the plaque biologically “high risk” and have been associated with increased rates of rupture, thrombosis, and cerebral embolization even in the absence of haemodynamically significant stenosis [2, 3].

In clinical practice, symptomatic status is often used to identify patients at elevated risk, yet a simple dichotomy between symptomatic and asymptomatic carotid stenosis is insufficient to capture the true spectrum of cerebrovascular vulnerability associated with carotid interventions. Increasing attention has been directed toward the presence, type, and volume of cerebral infarcts as additional markers of risk [4, 5]. In symptomatic patients, larger ischaemic lesion volumes have been associated with higher perioperative stroke risk after carotid endarterectomy (CEA) [4, 5], while in asymptomatic individuals, silent infarcts identify subgroups with significantly elevated long-term stroke risk [6], prompting consideration of earlier intervention [7]. Since the landmark clinical trials of carotid revascularization published in the 1990 s, major advances in best medical therapy [8] and surgical and endovascular techniques [9] have reshaped the risk-benefit profile of carotid revascularization, as illustrated by the recent findings of CREST-2 [10].

Beyond plaque-level pathology, the brain’s capacity to compensate for reduced extracranial inflow depends heavily on the configuration of the Circle of Willis (CW), the principal intracranial collateral network. Anatomical variations of the CW are common and may substantially modify susceptibility to cerebral ischaemia in carotid artery disease [11]. Incomplete or hypoplastic segments limit collateral recruitment and have been associated with impaired cerebral perfusion, higher rates of infarction, and worse stroke outcomes [12, 13].

Although plaque vulnerability, global extracranial carotid disease burden, and CW anatomy are each known to influence cerebrovascular risk, most prior studies have examined these factors in isolation and often in small or selected cohorts. Consequently, their combined contribution to the presence and pattern of ipsilateral cerebral infarcts—and their influence on long-term mortality—remains insufficiently defined in large, real-world CEA populations.

The aim of this study was to determine how CT angiography-derived carotid plaque morphology, bilateral carotid stenosis burden (carotid score), and CW anatomy jointly influence ipsilateral cerebral infarcts and long-term mortality in 1,304 consecutive CEA patients. We hypothesized that (1) plaque vulnerability features and compromised CW anatomy would independently predict ipsilateral cerebral infarcts; (2) watershed versus territorial infarcts would reflect predominantly hemodynamic versus embolic mechanisms, respectively; and (3) these vascular characteristics would be independently associated with long-term survival.

## Methods

### Study design and participants

This retrospective, single-center, observational cohort study included consecutive patients who underwent CEA between January 2013 and May 2018. Our study was approved by the Institutional Ethical Review Board (SERKEB 3/2026) and conducted in accordance with the Declaration of Helsinki. Indications for CEA followed contemporary guidelines [7].

**Fig. 1:**
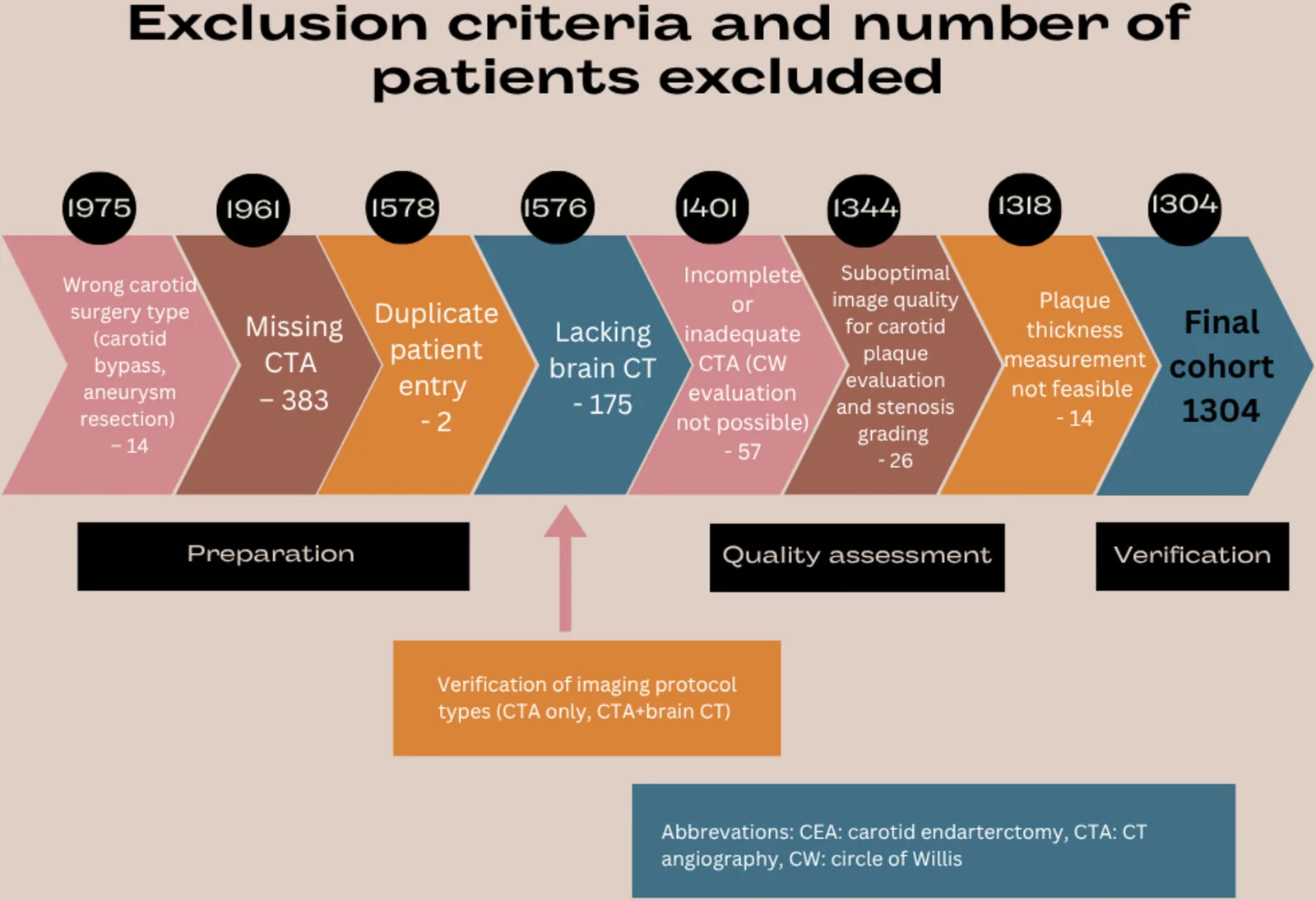
Flowchart demonstrating the exclusion criteria and number of patients excluded

Inclusion required adequate preoperative CT and CT angiography (CTA) to assess internal carotid artery (ICA) plaque morphology and CW anatomy. Inclusion and exclusion criteria are summarized in Table 1 and Fig. 1. Written informed consent was obtained prior to imaging. No patient selection was performed to avoid biases. Complete mortality follow-up was available through January 2025 via the Hungarian National eHealth Infrastructure.

**Table 1:** Inclusion and exclusion criteria of the study

Inclusion criteria	Exclusion criteria
Signed informed consent form	
≥ 70% (or 50–69% symptomatic) atherosclerotic stenosis of the extracranial internal carotid artery according to NASCET criteria on carotid CTA	
Complete CTA to evaluate the carotid and intracranial arteries	Missing or low quality CTA
Complete brain CT to evaluate cerebral infarcts	Missing or low quality brain CT

*CTA* CT angiography, *NASCET* North American Symptomatic Carotid Endarterectomy Trial

### CT and CTA acquisition

All patients underwent brain CT and carotid CTA using a 256-slice scanner (Brilliance iCT 256, Philips Healthcare), using acquisition parameters similar to those previously described [11].

Brain CT was performed with the following parameters: field of view 200–250 mm, collimation 64 × 0.625, pitch 0.39, gantry rotation time 400 ms, tube voltage 120 kVp, tube current 120–204 mAs, slice thickness 2 mm, dose-length product 312–626 mGycm).

CTA examinations were performed from the aortic arch to the vertex with the following parameters (field of view 180–200 mm, collimation 128 × 0.625, pitch 0.758, gantry rotation time 330 ms, tube voltage 120 kVp, tube current 76–206 mAs, dose-length product 220–608 mGycm). Bolus tracking technique in the aortic arch was used with 50 ml of iodinated contrast agent (Iomeron 400, Bracco Imaging SpA, Milan, Italy) followed by 40 ml saline bolus, injected at 5 ml/s through a 18G cannula. Contiguous sections were reconstructed with 0.67-mm slice thickness and 512 × 512 matrix using iterative model reconstruction (IMR, Philips Healthcare, Cleveland, OH, USA). A dedicated workstation was used for image interpretation (IntelliSpace Portal, Philips Healthcare, Best, The Netherlands) with interactive multiplanar reformats to evaluate the CW anatomy and plaque features. Advanced Vascular Analysis software was utilized to study the internal carotid (ICA) and vertebral arteries (VA) with curved reformations.

### Brain infarct detection and classification

**Fig. 2:**
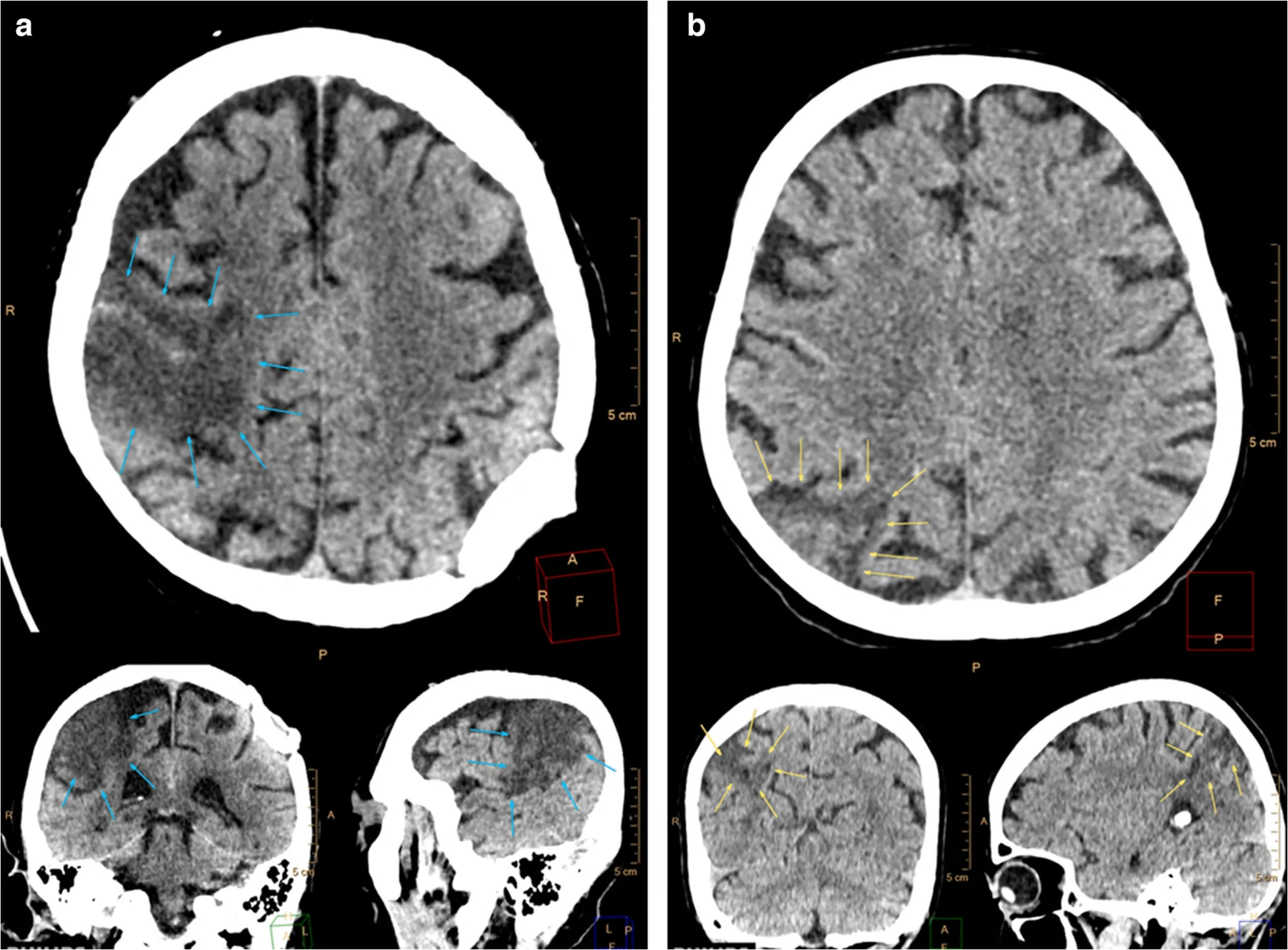
**a**, **b** Multiplanar CT reformats of **a** voluminous ill-defined cortical-subcortical hypodensity in the right frontal and parietal lobes (*blue arrows*), corresponding to a subacute middle cerebral artery territorial infarct of a patient with an exulcerated plaque and a carotid score of 4 (70% stenosis of the right internal carotid artery and no stenosis of the contralateral internal carotid artery). Left lateral craniectomy following surgical removal of an old meningioma; **b** ill-defined cortical-subcortical hypodensity in the right upper parietal lobe (*yellow arrows*), corresponding to a subacute triple watershed infarct of a patient with a carotid score of 1 (˃90% stenosis of the right internal carotid artery caused by a calcified plaque and 70% stenosis of the contralateral internal carotid artery)

Brain infarcts were determined as definitive hypodens lesions corresponding to the usual anatomy of the anterior circulation (i.e. ICA territory) or of watershed areas of the ipsilateral cerebral hemisphere by two experienced radiologists (20 and 5 years of experience). With foetal-type ipsilateral posterior cerebral artery, originating from the ICA with a non-visualized or hypoplastic precommunicating segment, posterior circulation infarcts were defined as relevant infarcts, as well. Cortical-subcortical and basal ganglia infarcts were considered as territorial infarcts (Fig. 2a), whereas infarcts between vascular territories, as watershed infarcts (anterior, posterior, triple, and middle, i.e. in between the pial and deep branches of the middle cerebral artery). Figure 2b. The number and type of infarcts were recorded in each patient, and two patient groups were determined (territorial or watershed), according to the infarct type (if a single lesion was present) or the dominant infarct type (in case of mixed territorial and watershed infarcts). We dichotomized patients as ones with or without infarcts for a simplified analysis.

### Carotid stenosis assessment and carotid score

ICA stenosis was measured on both sides according to the North American Symptomatic Carotid Endarterectomy Trial (NASCET) method [1] and categorized: < 50%, 50–69%, 70–89%, 90–99%, and occluded. A carotid score (0–4) was developed to reflect global extracranial carotid disease burden, with lower scores indicating more advanced bilateral disease:

- 0: contralateral ICA occlusion, independent of the stenosis degree of the operated ICA,
- 1: > 90% ipsilateral and 70–99% contralateral ICA stenosis,
- 2: > 90% ipsilateral and 0–69% contralateral ICA stenosis,
- 3: 50–89% ipsilateral, and 70–99% contralateral ICA stenosis,
- 4: 50–89% ipsilateral, and 0–69% contralateral ICA stenosis.

Twenty-six cases lacked precise measurements due to suboptimal CTA quality.

### Plaque characterization and plaque thickness measurements

**Fig. 3:**
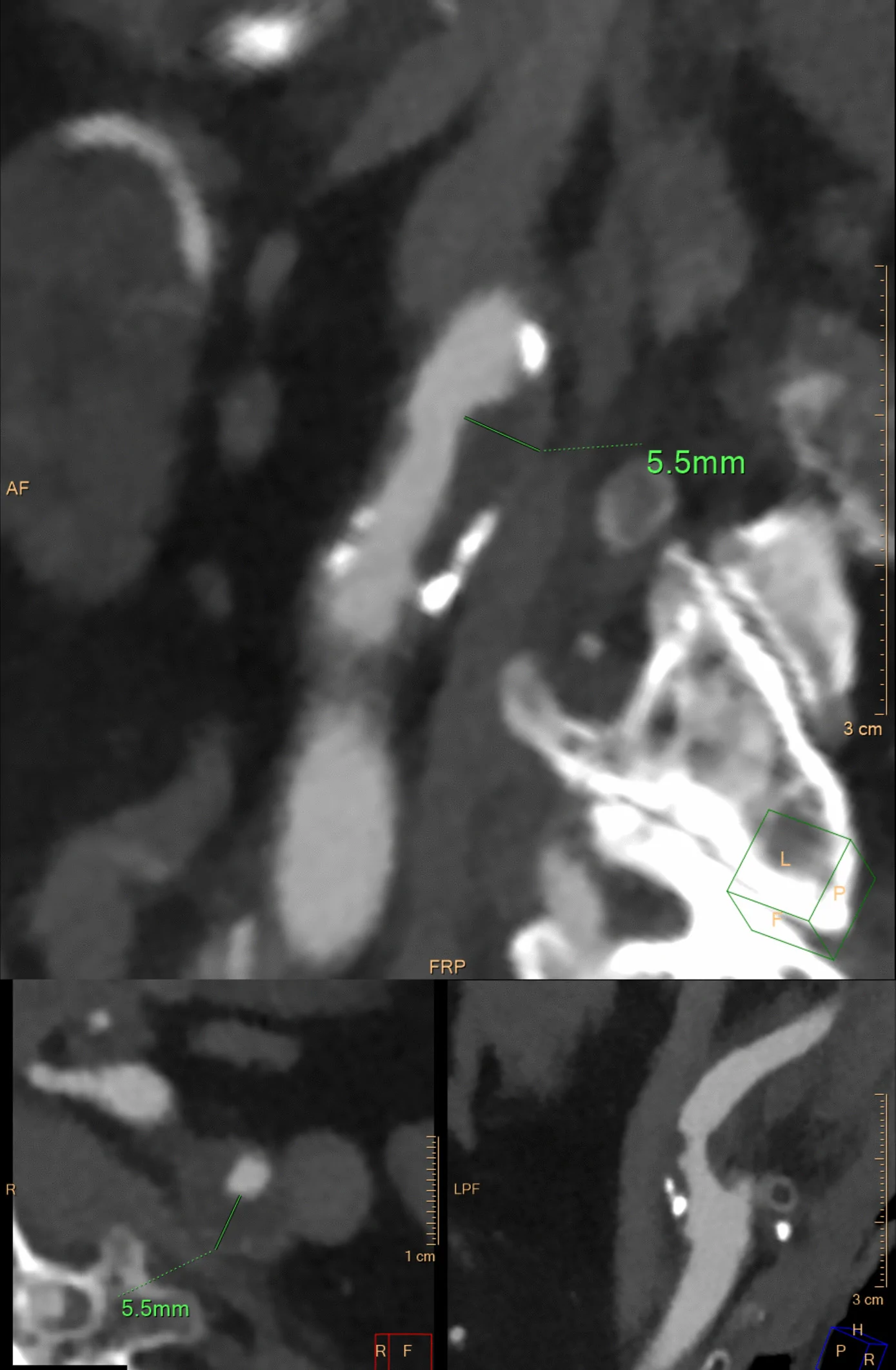
Multiplanar CT reformats perpendicular to the residual lumen of the stenosed right internal carotid artery, with measurement at the site of maximal plaque thickness (*green text*)

**Fig. 4:**
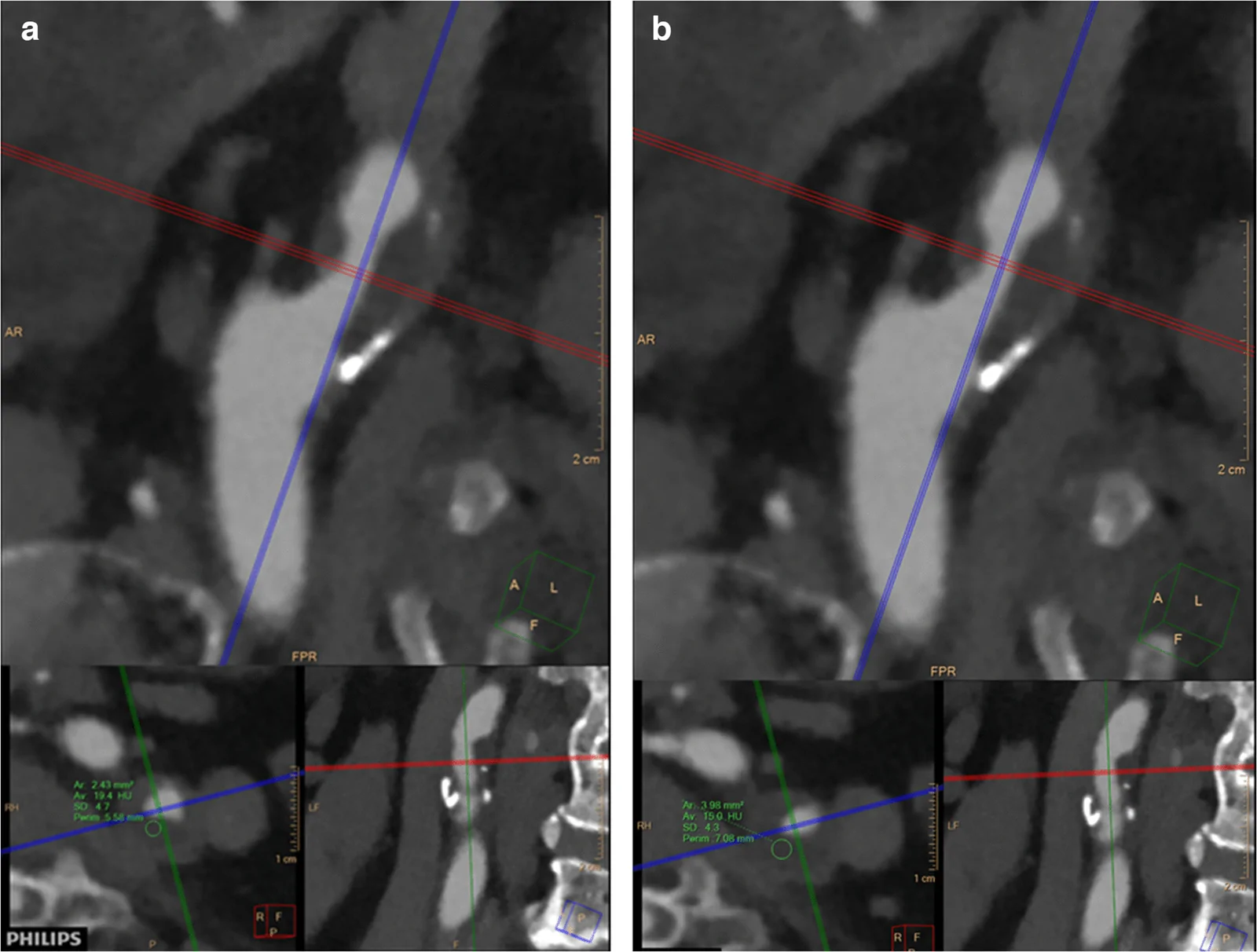

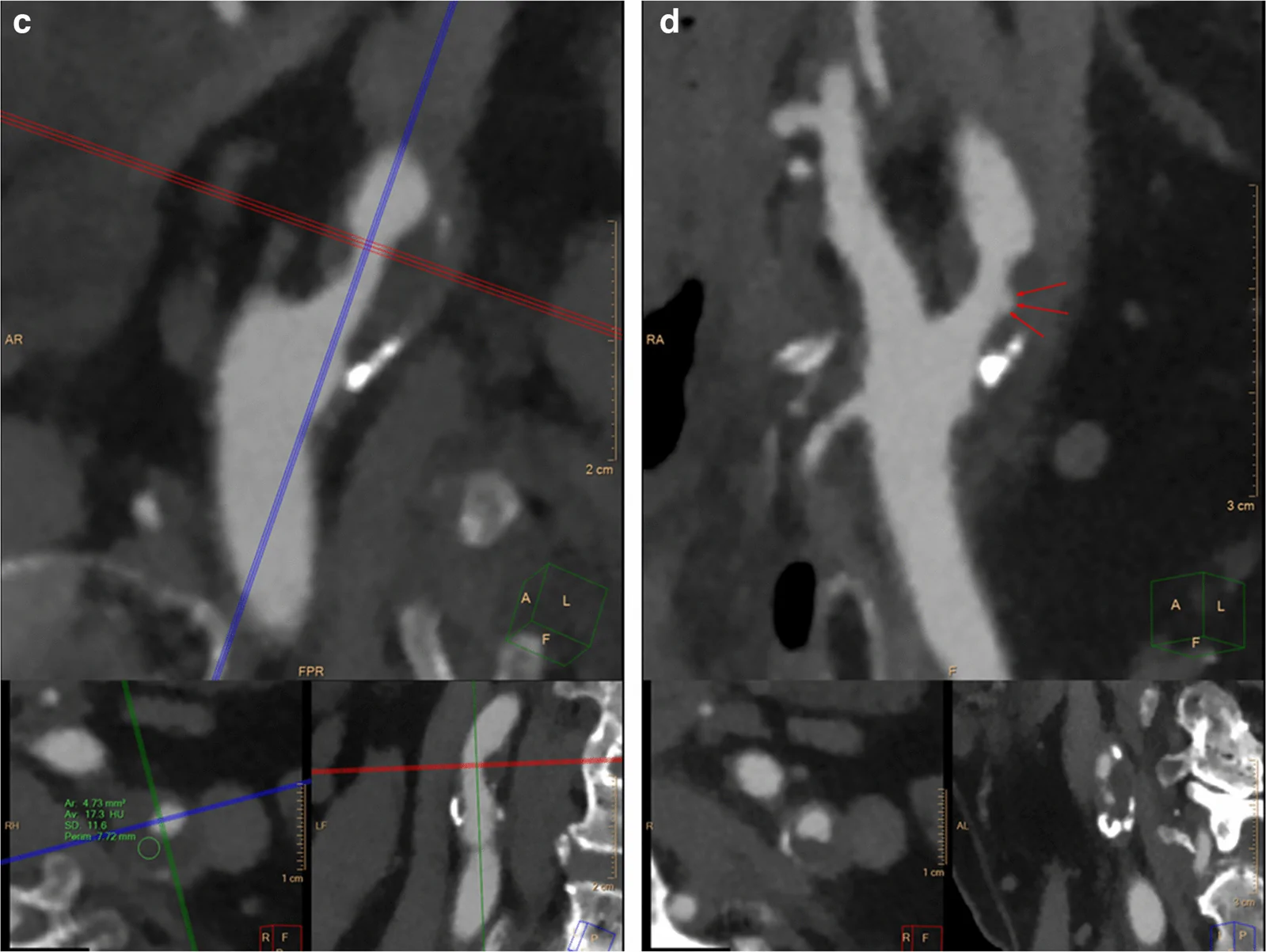
**a**–**d** Multiplanar CT reformats perpendicular to the residual lumen of the stenosed right internal carotid artery **a**–**c** with plaque density measurements (*green regions of interest*) in three consecutive axial slices, showing a dominantly lipid-rich plaque with an average density of 15–19 Hounsfield Units.** d** Contrast filling (*red arrows*) pointing at an exulceration within a dominantly lipid-rich plaque

Multiplanar and curved reconstructions were used to determine maximal plaque thickness (Fig. 3) and minimal residual lumen. Carotid bifurcation plaques were analyzed qualitatively: calcified if > 120 HU, fibrotic if 60–120 HU, lipid-rich if < 60 HU [14, 15], exulcerated after measuring/averaging plaque densities in three consecutive axial slices at maximum plaque thickness (Fig. 4a–c). Exulcerated plaques were defined as apparent contour irregularities or excavations within a plaque (˃2 mm) filled with contrast material (Fig. 3d). The measurements were performed by two experienced vascular radiologists in consensus. Additionally, interreader agreement was assessed in a subset of 100 randomly selected cases, which were independently evaluated by a third reader (post-doctoral scholar with 7 years of radiology experience). The third reader applied the same predefined, literature-based criteria for plaque characterization as used in the primary analysis.

### CW analysis

**Fig. 5:**
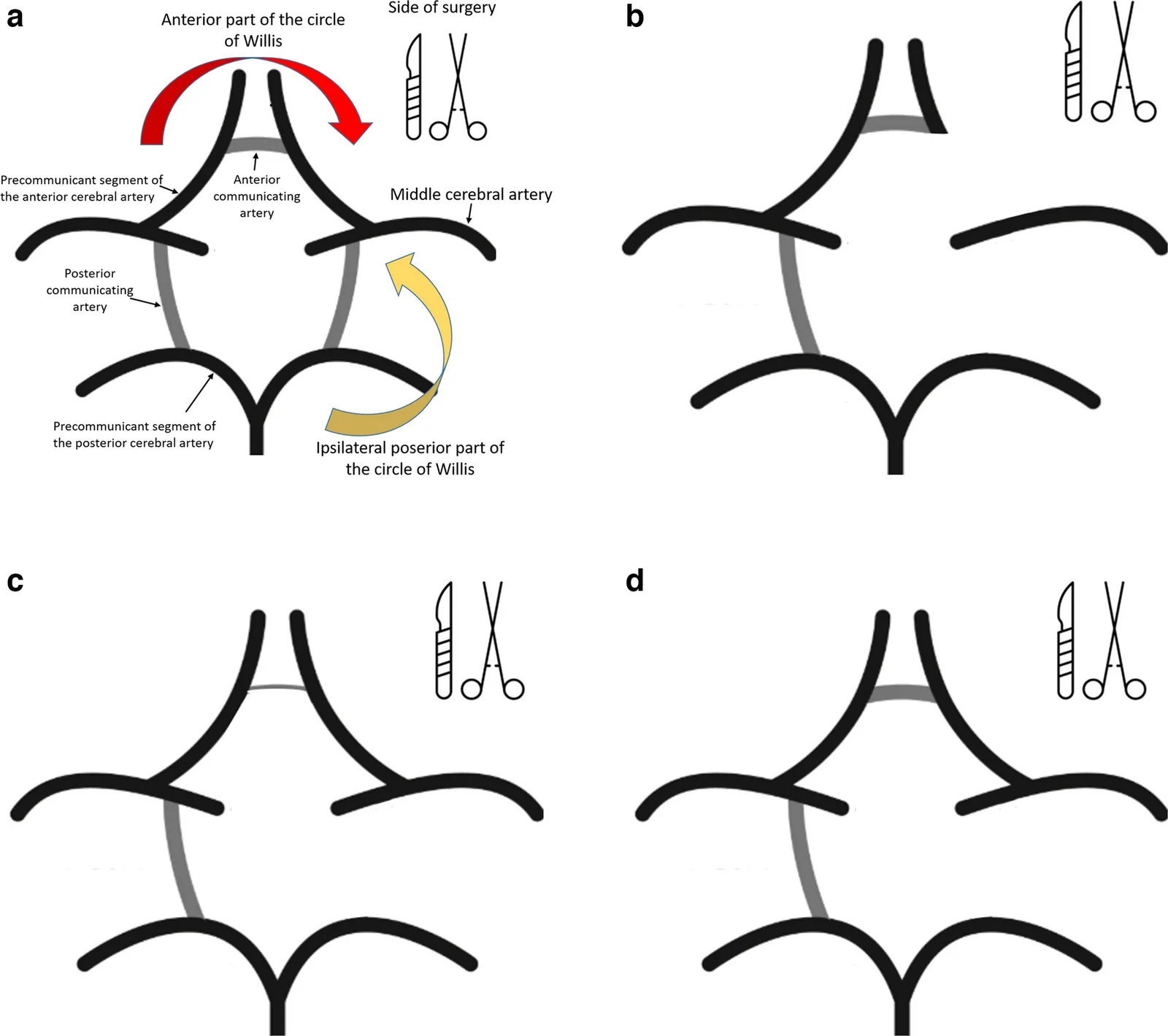

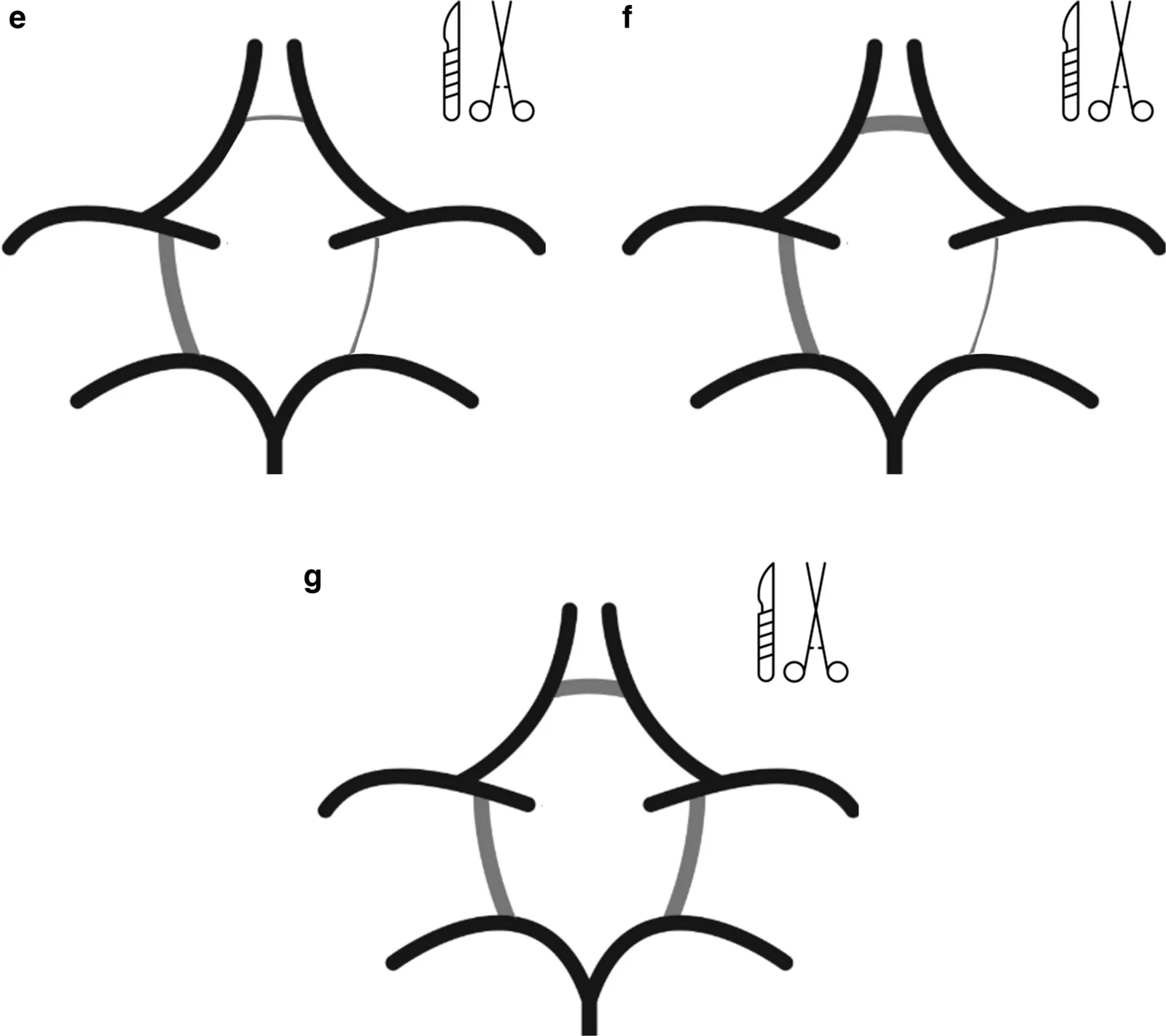
**a**–**g** Schematic illustration of the circle of Willis (CW) types; **a** Normal CW segments, demonstrating collateral supply from the anterior (curved thick red arrow) and ipsilateral posterior parts (curved thick yellow arrow) to the index middle cerebral artery during surgery; **b** Type 0 (severely compromised) CW: both the anterior and ipsilateral posterior parts are incomplete. Non-visualisation of the ipsilateral anterior precommunicating segment and ipsilateral posterior communicating artery was the most common variant; **c** Type 1 (severely compromised) CW: one part incomplete, the other hypoplastic. Hypoplasia of the anterior communicating artery and non-visualisation of the ipsilateral posterior communicating artery was the most frequent; **d** Type 2 CW: one part incomplete, the other normal. Non-visualisation of the ipsilateral posterior communicating artery was the most common variant; **e** Type 3 CW: both the anterior and ipsilateral parts hypoplastic. Hypoplasia of the anterior and ipsilateral posterior communicating arteries was the most frequent; **f** Type 4 CW: one of the parts hypoplastic. Hypoplasia of the ipsilateral posterior communicating artery was the most common variant; **g** Type 5 CW: both the anterior and ipsilateral posterior parts normal

Each CW segment was classified as normal (diameter ≥ 0.8 mm), hypoplastic (< 0.8 mm) or non-visualized. The anterior and the ipsilateral posterior parts of the CoW were considered separately and grouped as complete (all segments normal, Fig. 5a), hypoplastic (any of the components hypoplastic) or incomplete (any of the segments non-visualized). Then we reclassified our subjects into CW groups as follows:

- Type 0: both the anterior and ipsilateral posterior parts incomplete (multiple incompleteness of the CW) (Fig. 5b)
- Type 1: one part incomplete, the other hypoplastic (Fig. 5c)
- Type 2: one part incomplete, the other normal (Fig. 5d)
- Type 3: both the anterior and ipsilateral parts hypoplastic (Fig. 5e)
- Type 4: one of the two parts hypoplastic (Fig. 5f)
- Type 5: both the anterior and ipsilateral posterior parts normal (Fig. 5g)

**Fig. 6:**
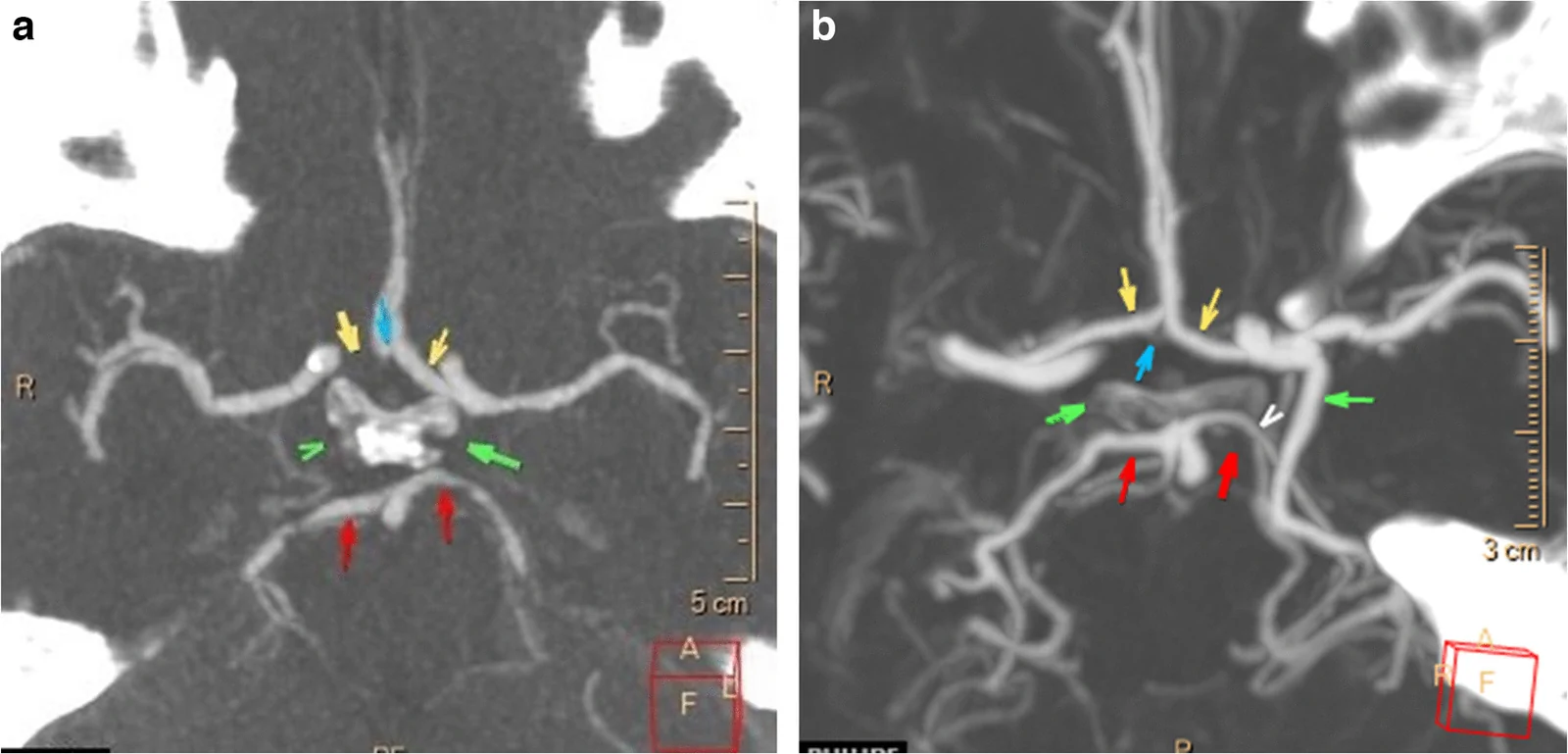
**a**, **b** Thick slab (14 mm) maximal intensity projections of **a** type 0 circle of Willis in a patient with left internal carotid artery stenosis. Non-visualization of the right precommunicating segment of the anterior cerebral artery (*double yellow arrows*). Well-developped anterior communicating artery (*blue arrow*). Normal left precommunicating segment of the anterior cerebral artery (*yellow arrow*). Non-visualization of the ipsilateral (left) posterior communicating artery (*double green arrows*). Normal precommunicating segment of the posterior cerebral arteries (*red arrows*). Hypoplasia of the contralateral posterior communicating artery, poorly visualized (*green arrowhead*); **b** type 1 circle of Willis in a patient with left internal carotid artery stenosis. Hypoplasia of the anterior communicating artery (*blue arrow*). Non-visualization of the ipsilateral (left) precommunicating segment of the posterior cerebral artery (*double red arrows*). Well-developped ipsilateral posterior communicating artery (*green arrow*). Non-visualization of the contralateral posterior communicating artery (*double green arrows*). Precommunicating segment of the anterior cerebral arteries (yellow arrows). The left superior cerebellar artery follows the course of the missing left precommunicating segment (*white arrowhead*). For explanation of circle of Willis types refer to text

The most severe constellation was incompleteness of both the anterior and ipsilateral posterior parts feeding the middle cerebral artery of the operated side. Types 0–1 were defined as severely compromised CW anatomy, representing major limitations of collateral flow (Fig. 6a, b).

Given their rarity and limited statistical power, cases with major vertebrobasilar abnormalities potentially affecting collateralization were excluded from CW-related analyses.

### Mortality data

All-cause mortality was extracted from the Hungarian National eHealth Infrastructure in January 2025. Cause-specific mortality data were unavailable due to incomplete national coding; therefore, analyses focused on overall mortality.

### Statistical analysis

All analyses were performed using IBM SPSS Statistics, version 30.0 (IBM Corp., Chicago, IL). Continuous variables are presented as mean ± standard deviation or median (interquartile range), as appropriate. Categorical variables are reported as absolute counts and frequencies. Group comparisons were performed using the two-sample t-test for continuous variables and the chi-squared test for categorical variables. Agreement for plaque composition classification was evaluated using Cohen’s kappa, while reproducibility of plaque thickness measurements was assessed using a two-way mixed-effects intraclass correlation coefficient (ICC). A logistic regression analysis was conducted to ascertain the predictors of the presence of brain infarcts and territorial versus watershed infarct patterns. Predictors of all-cause mortality were identified using Cox proportional-hazards models. For all multivariable analyses, variables that demonstrated significant association (*p* < 0.05) in univariable analysis were entered into the multivariable models. The statistical significance of the results was determined by a two-sided p-value less than 0.05.

## Results

### Study population

During the study period, 1,975 carotid endarterectomies were performed. After applying inclusion and exclusion criteria, 1,304 patients were included in the analysis (Fig. 1). Mean age was 68.6 ± 8.0 years, and 38.3% were female. Overall, 416 patients (31.9%) were symptomatic, including 192 (14.7%) with prior stroke, 163 (12.5%) with transient ischemic attack, and 61 (4.7%) with amaurosis fugax. Transient ischemic attack was defined as a focal neurological deficit lasting less than 24 h, stroke was defined as a focal neurological deficit with a duration of more than 24 h. Amaurosis was defined as a retinal deficit, either transient or permanent.

### Description of radiological characteristics

#### Prevalence and pattern of cerebral infarcts

Definitive ipsilateral cerebral infarcts were identified in 479 of 1,304 patients (36.7%). Of these, 265 patients (20.3%) had predominantly territorial infarcts, whereas 214 (16.4%) had predominantly watershed infarcts. The distribution of infarct subtypes is detailed in Table 2.

**Table 2:** Rate of different infarct types in 479 carotid endarterectomy subjects with brain infarcts

	Brain infarcts total	(Dominantly) Territorial		(Dominantly) Watershed	
	*N* = 479 (36.7)	*N* = 265 (20.3)		*N* = 214 (16.4)	
Single	225 (47.0)	144 (54.3)		81 (37.9)	
Multiple	254 (53.0)	121 (45.7)		133 (62.1)	
		Multiple purely territorial	Mixed territorial ˃ watershed	Multiple purely watershed	Mixed watershed ˃ territorial
		61 (50.4)	60 (49.6)	53 (39.8)	80 (60.2)

Categorical variables shown as numbers (percentages)

#### Carotid stenosis severity and carotid score

Ninety-four patients eligible for CEA had contralateral ICA occlusion (carotid score 0). Eighty-eight subjects had > 90% ipsilateral and 70–99% contralateral ICA stenosis (score 1). Three hundred ninety-eight subjects had > 90% ipsilateral and 0–69% contralateral ICA stenosis (score 2), One hundred three had 50–89% ipsilateral, and 70–99% contralateral ICA stenosis (score 3). The largest subgroup consisted of 595 patients with study participants having 50–89% ipsilateral, and 0–69% contralateral ICA stenosis (score 4). In twenty-six cases, precise grading was not possible due to suboptimal CTA quality.

Major vertebrobasilar abnormalities were uncommon (bilateral vertebral or basilar artery non-visualization/occlusion in 10 cases; combination of vertebral artery hypoplasia/significant stenosis and non-visualization/occlusion of the other vertebral artery in 19; bilateral vertebral and/or basilar artery hypoplasia/significant stenosis in 22), and only 1 out of 51 was associated with a severely compromised CW (type 0); therefore, these cases were excluded from CW-related analyses.

#### Plaque thickness and plaque morphology

Mean maximal ipsilateral plaque thickness was 5.0 ± 1.3 mm (range 2.0–10.1 mm). Patients with cerebral infarcts had significantly thicker plaques than those without infarcts (5.2 ± 1.4 mm vs 4.9 ± 1.2 mm, *p* < 0.001; Table 3).

**Table 3:** Demographic data and cardiovascular risk profile of patients with and without ipsilateral brain infarcts

	Total	Infarct–	Infarct +	*p*
	*N* = 1304	*N* = 825	*N* = 479	
Age (years)	68.6 ± 8.0	68.5 ± 7.7	68.9 ± 8.6	0.40
Female sex,* n* (%)	500 (38.3)	326 (39.5)	174 (36.3)	0.25
*Symptomatic, n (%)*	***416 (31.9)***	***183 (22.2)***	***233 (48.6)***	** < *0.001***
Cardiovascular risk factors				
Coronary artery disease, *n* (%)	421 (32.3)	276 (33.5)	145 (30.3)	0.24
Hypertension, *n* (%)	1186 (91.0)	750 (90.9)	436 (91.0)	0.75
Dyslipidemia,* n* (%)	498 (38.2)	314 (38.1)	184 (38.4)	0.90
Chronic obstructive pulmonary disease, *n* (%)	152 (11.7)	96 (11.6)	56 (11.7)	0.98
Severe chronic kidney disease, *n* (%)	48 (3.7)	27 (3.3)	21 (4.4)	0.30
Current smoking, *n* (%)	472 (36.2)	296 (35.9)	176 (36.7)	0.75
Diabetes, *n* (%)	451 (34.6)	282 (34.2)	169 (35.3)	0.69
Peripheral artery disease, *n* (%)	260 (19.9)	168 (20.4)	92 (19.2)	0.61
Aortic disease (aneurysm/dissection), *n* (%)	32 (2.5)	17 (2.1)	15 (3.1)	0.27
*Carotid score*	***2.8* ± *1.3***	***2.9* ± *1.3***	***2.6* ± *1.3***	** < *0.001***
*Ipsilateral carotid plaque thickness (mm)*	***5.0* ± *1.3***	***4.9* ± *1.2***	***5.2* ± *1.4***	** < *0.001***
Ipsilateral CW anatomy				
*Multiple incompleteness (Type 0)*	***81(6.2)***	***42 (5.1)***	***39 (8.1)***	***0.03***
*One part incomplete, other hypoplastic*	***226 (17.3)***	***128 (15.5)***	***98 (20.5)***	***0.02***
One part incomplete, other normal	306 (23.5)	194 (23.5)	112 (23.4)	0.96
Both parts hypoplastic	125 (9.6)	80 (9.7)	45 (9.4)	0.86
One part hypoplastic, other normal	352 (27.0)	230 (27.9)	122 (25.5)	0.34
*Both parts normal*	***214 (16.4)***	***151 (18.3)***	***63 (13.2)***	***0.02***
Plaque type				
Calcified	379 (29.1)	254 (30.8)	125 (26.1)	0.07
*Fibrotic*	***175 (13.4)***	***125 (15.2)***	***50 (10.4)***	***0.02***
Lipid-rich	455 (34.9)	292 (35.4)	163 (34.0)	0.62
*Exulcerated*	***295 (22.6)***	***154 (18.7)***	***141 (29.4)***	** < *0.001***

Continuous data are presented as mean ± standard deviation, while categorical variables are shown as numbers and percentages, *CW* circle of Willis

Plaque composition was categorized as calcified in 29.1%, fibrotic in 13.4%, lipid-rich in 34.9%, and exulcerated in 22.6% of patients. Lipid-rich and exulcerated plaques together accounted for 57.5% of lesions and were considered high-risk plaque types.

Interreader agreement for the classification of plaque composition was substantial (κ=0.68; 95%CI: 0.57–0.80), with good reproducibility for plaque thickness measurements (ICC=0.85; 95%CI: 0.78–0.90).

#### Circle of Willis anatomy

Severely compromised CW anatomy (types 0–1) was present in 307/1,304 patients (23.5%). Type 0 CW (multiple incompleteness of the ipsilateral CW) was encountered in 81 (6.2%) subjects, including non-visualization of the precommunicating segment of the right/left anterior cerebral artery in 62, or non-visualization of the anterior communicating artery in 19 cases. The incompleteness of the posterior part was secondary to the non-visualization of the precommunicating segment of the ipsilateral posterior cerebral artery in 13, or of the ipsilateral posterior communicating artery in 68 cases.

Type 1 CW, the combination of hypoplasia and incompleteness accounted for 226 (17.3%) cases, with incompleteness of the anterior part in 32 and of the posterior part in the remaining 194 subjects (194/226, 85.8%).

Type 2 CW was found in 306 (23.5%)—mostly the posterior part was incomplete 274/306 (89.9%). Type 3 CW was detected in 125 (9.6%), type 4 in 352 (27.0%) of subjects, again the posterior part was more prone to hypoplasia (223/352, 63.3%). As for the anterior part, the most common was the hypoplasia of the anterior communicating artery (74/352, 21.0%).

A completely normal CW (type 5) was present in 214 participants (16.4%). Detailed distributions of CW types are shown in Table 4.

**Table 4:** Anatomic variants of circle of Willis and their components

*N* = 1304	Number	Anterior part of the CW	Ipsilateral posterior part of the CW
Type 0 *n* = 81 (6.2)	52	**Non-visualised precommunicating segment right/left anterior cerebral artery**	**Non-visualised posterior communicating artery**
	16	**Non-visualised anterior communicating artery**	**Non-visualised posterior communicating artery**
	10	**Non-visualised precommunicating segment right/left anterior cerebral artery**	**Non-visualised precommunicating segment right/left posterior cerebral artery**
	3	**Non-visualised anterior communicating artery**	**Non-visualised precommunicating segment right/left posterior cerebral artery**
Type 1 *n* = 226 (17.3)	112	*Hypoplasia of anterior communicating artery*	**Non-visualised posterior communicating artery**
	59	*Hypoplasia of precommunicating segment right/left anterior cerebral artery*	**Non-visualised posterior communicating artery**
	20	**Non-visualised precommunicating segment right/left anterior cerebral artery**	*Hypoplasia of posterior communicating artery*
	9	*Hypoplasia of anterior communicating artery*	**Non-visualised precommunicating segment right/left posterior cerebral artery**
	9	*Hypoplasia of precommunicating segment right/left anterior cerebral artery*	**Non-visualised precommunicating segment right/left posterior cerebral artery**
	9	**Non-visualised precommunicating segment right/left anterior cerebral artery**	*Hypoplasia of precommunicating segment right/left posterior cerebral artery*
	4	**Non-visualised anterior communicating artery**	*Hypoplasia of posterior communicating artery*
	4	**Non-visualised anterior communicating artery**	*Hypoplasia of precommunicating segment right/left posterior cerebral artery*
Type 2 *n* = 306 (23.5)	250	Normal anterior CW segments	**Non-visualised posterior communicating artery**
	24	Normal anterior CW segments	**Non-visualised precommunicating segment right/left posterior cerebral artery**
	23	**Non-visualised precommunicating segment right/left anterior cerebral artery**	Normal posterior CW segments
	9	**Non-visualised anterior communicating artery**	Normal posterior CW segments
Type 3 *n* = 125 (9.6)	59	*Hypoplasia of anterior communicating artery*	*Hypoplasia of posterior communicating artery*
	42	*Hypoplasia of precommunicating segment right/left anterior cerebral artery*	*Hypoplasia of posterior communicating artery*
	12	*Hypoplasia of precommunicating segment right/left anterior cerebral artery*	*Hypoplasia of precommunicating segment right/left posterior cerebral artery*
	12	*Hypoplasia of anterior communicating artery*	*Hypoplasia of precommunicating segment right/left posterior cerebral artery*
Type 4 *n* = 352 (27)	193	Normal anterior CW segments	*Hypoplasia of posterior communicating artery*
	72	*Hypoplasia of anterior communicating artery*	Normal posterior CW segments
	53	*Hypoplasia of precommunicating segment right/left anterior cerebral artery*	Normal posterior CW segments
	34	Normal anterior CW segments	*Hypoplasia of precommunicating segment right/left posterior cerebral artery*
Type 5 *n* = 214 (16.4)	214	Normal anterior CW segments	Normal posterior CW segments

Categorical variables shown as numbers (percentages), *CW* circle of Willis

### Associations with ipsilateral cerebral infarcts

Patients with cerebral infarcts were more frequently symptomatic (48.6% vs 22.2%, *p* < 0.001), had lower carotid scores (2.6 ± 1.3 vs 2.9 ± 1.3, *p* < 0.001), and exhibited greater plaque thickness, compared to those without an infarct (5.2 ± 1.4 mm vs 4.9 ± 1.2 mm, *p* < 0.001). The two groups did not differ significantly regarding demographic data or main cardiovascular risks. Considering CW anatomy, those subjects with infarcts showed a significantly higher rate of severely compromised CW (multiple incompleteness: 8.1% vs 5.1%, *p* = 0.03; one part incomplete, other hypoplastic: 20.5% vs 15.5%, *p* = 0.02). Moreover, there was a higher percentage of normal CW in patients without brain infarcts compared to those with infarcts (18.3% vs 13.2%, *p* = 0.02). Infarct patients presented more frequently with exulcerated plaques (29.4% vs 18.7%, *p* < 0.001), and less frequently with fibrotic plaques (10.4% vs 15.2%, *p* = 0.02).

In univariable analysis, symptomatic stenosis status (Odds ratio (OR): 3.32, 95% confidence interval (CI): 2.61–4.24, *p* < 0.001), thicker ipsilateral carotid plaques (OR: 1.18, 95%CI: 1.08–1.28, *p* < 0.001), exulcerated plaques (OR: 1.61, 95%CI: 1.20–2.15, *p* = 0.001), multiple incompleteness of the ipsilateral CW (OR: 1.65, 95%CI: 1.05–2.60, *p* = 0.03), combined incompleteness and hypoplasia of the ipsilateral CW (OR: 1.40, 95%CI: 1.05–1.87, *p* = 0.02) predicted ipsilateral brain infarcts (Table 5). Higher carotid scores reflecting lower-grade stenoses (OR: 0.82, 95%CI: 0.75–0.89, *p* < 0.001) and normal CW configuration (OR: 0.68, 95%CI: 0.49–0.93, *p* = 0.02) were protective. Similarly, fibrotic plaques showed an inverse association with brain infarcts (OR: 0.65, 95%CI: 0.46–0.93, *p* = 0.02).

**Table 5:** Determinants of the presence of ipsilateral brain infarcts

	Univariable			Multivariable		
	OR	95% CI	*p*	OR	95% CI	*p*
Age	1.01	0.99–1.02	0.40			
Female sex	0.87	0.69–1.10	0.25			
*Symptomatic*	***3.32***	***2.61–4.24***	** < *0.001***	***3.20***	***2.48–4.12***	** < *0.001***
Cardiovascular risk factors						
Coronary artery disease	1.16	0.91–1.48	0.24			
Hypertension	0.99	0.67–1.46	0.94			
Dyslipidemia	0.99	0.78–1.24	0.90			
Chronic obstructive pulmonary disease	0.99	0.70–1.41	0.98			
Severe chronic kidney disease	0.74	0.41–1.32	0.31			
Current smoking	0.96	0.76–1.22	0.75			
Diabetes	0.95	0.75–1.21	0.69			
Peripheral artery disease, *n* (%)	0.93	0.70–1.23	0.69			
Aortic disease (aneurysm/dissection),* n* (%)	1.54	0.76–3.11	0.23			
*Carotid score*	***0.82***	***0.75–0.89***	** < *0.001***	***0.81***	***0.74–0.89***	** < *0.001***
*Ipsilateral carotid plaque thickness (mm)*	***1.18***	***1.08–1.28***	** < *0.001***	***1.12***	***1.02–1.24***	***0.02***
Ipsilateral CW anatomy						
*Multiple incompleteness (Type 0)*	***1.65***	***1.05–2.60***	***0.03***	***2.06***	***1.26–3.36***	***0.004***
*One part incomplete, other hypoplastic*	***1.40***	***1.05–1.87***	***0.02***	***1.54***	***1.11–2.12***	***0.01***
One part incomplete, other normal	0.99	0.76–1.30	0.96			
Both parts hypoplastic	0.97	0.66–1.42	0.86			
One part hypoplastic, other normal	0.88	0.68–1.14	0.34			
*Both parts normal*	***0.68***	***0.49–0.93***	***0.02***	0.73	0.51–1.04	0.08
Plaque type						
Calcified	0.79	0.62–1.02	0.07			
*Fibrotic*	***0.65***	***0.46–0.93***	***0.02***	0.85	0.58–1.25	0.40
Lipid-rich	0.94	0.74–1.19	0.62			
*Exulcerated*	***1.82***	***1.40–2.37***	** < *0.001***	***1.61***	***1.20–2.15***	***0.001***

*CW* circle of Willis

In multivariable analysis, symptomatic status (OR: 3.20, 95%CI: 2.48–4.12, *p* < 0.001), carotid plaque thickness (OR:1.12, 95%CI: 1.02–1.24, *p* = 0.02), multiple incompleteness of the ipsilateral CW (OR: 2.06, 95%CI: 1.26–3.36, *p* = 0.004), combined incompleteness and hypoplasia of the ipsilateral CW (OR: 1.54, 95%CI: 1.11–2.12, *p* = 0.01) and an inverse association with carotid score (OR 0.81, 95% CI 0.74–0.89; *p* < 0.001) remained independent predictors of brain ischaemia. 

### Determinants of infarct pattern

In univariable analysis, lower carotid scores predicted watershed rather than territorial infarcts (OR 0.83, 95% CI 0.75–0.92; *p* < 0.001), whereas symptomatic status (OR 2.49, 95% CI 1.89–3.28; *p* < 0.001), greater plaque thickness (OR 1.14, 95% CI 1.03–1.27; *p* = 0.01), and exulcerated plaques (OR 1.54, 95% CI 1.14–2.09; *p* = 0.02) were associated with territorial infarcts (Table 6).

**Table 6:** Predictors of territorial (1) vs. watershed (0) infarcts

	Univariable			Multivariable		
	OR	95% CI	*p*	OR	95% CI	*p*
Age	1.00	0.98–1.02	0.92			
Female sex	0.81	0.61–1.07	0.13			
*Symptomatic*	***2.49***	***1.89–3.28***	** < *0.001***	***2.29***	***1.72–3.04***	** < *0.001***
Cardiovascular risk factors						
Coronary artery disease	1.16	0.86–1.55	0.34			
Hypertension	0.94	0.59–1.52	0.94			
Dyslipidemia	1.16	0.87–1.53	0.31			
Chronic obstructive pulmonary disease	0.95	0.63–1.44	0.81			
Severe chronic kidney disease	0.85	0.43–1.70	0.65			
Current smoking	1.18	0.89–1.57	0.26			
Diabetes	1.04	0.78–1.38	0.81			
*Carotid score*	***0.83***	***0.75–0.92***	** < *0.001***	***0.85***	***0.76–0.94***	***0.02***
*Carotid plaque thickness (mm)*	***1.14***	***1.03–1.27***	***0.01***	1.10	0.98–1.22	0.10
Ipsilateral CW anatomy						
Multiple incompleteness (Type 0)	1.31	0.78–2.21	0.31			
One part incomplete, other hypoplastic	1.11	0.78–1.57	0.58			
One part incomplete, other normal	1.08	0.79–1.47	0.65			
Both parts hypoplastic	0.98	0.62–1.55	0.93			
One part hypoplastic, other normal	1.01	0.75–1.37	0.95			
Both parts normal	0.70	0.47–1.04	0.08			
Plaque type						
Calcified	0.91	0.67–1.23	0.54			
Fibrotic	0.72	0.47–1.10	0.13			
Lipid-rich	0.89	0.67–1.19	0.43			
*Exulcerated*	***1.54***	***1.14–2.09***	***0.01***	***1.38***	***1.01–1.90***	***0.047***

*CW* circle of Willis

In multivariable analysis, lower carotid score remained an independent predictor of watershed infarcts (OR 0.85, 95% CI 0.76–0.94; *p* = 0.02), while symptomatic status (OR 2.29, 95% CI 1.72–3.04; *p* < 0.001) and exulcerated plaques (OR 1.38, 95% CI 1.01–1.90; *p* = 0.047) independently predicted territorial infarcts.

### Long-term mortality

Of the 1304 patients included in the study, 636 (48.8%) died during follow-up. The mean survival time from surgery to death was 54.9 months, with a range of 6 days to 10.9 years. In univariable analysis, increasing age, male sex, and the presence of ipsilateral cerebral infarcts were strong predictors of overall mortality (Table 7). Age was associated with higher mortality both in univariable (HR 1.05, 95% CI 1.04–1.06; *p* < 0.001) and multivariable analyses (HR 1.04, 95% CI 1.03–1.05; *p* < 0.001). Female sex was inversely associated with mortality (univariable HR 0.80, 95% CI 0.68–0.94; *p* = 0.01; multivariable HR 0.77, 95% CI 0.64–0.91; *p* = 0.003). The presence of ipsilateral brain infarcts was a particularly strong determinant of mortality in both univariable (HR 1.52, 95% CI 1.30–1.78; *p* < 0.001) and multivariable analyses (HR 1.49, 95% CI 1.27–1.75; *p* = 0.003) (Table 7).

**Table 7:** Predictors of mortality assessed by Cox regression models

	Univariable			Multivariable		
	HR	95% CI	*p*	HR	95% CI	*p*
*Age*	***1.05***	***1.04–1.06***	** < *0.001***	***1.04***	***1.03–1.05***	** < *0.001***
*Female sex*	***0.80***	***0.68–0.94***	***0.01***	***0.77***	***0.64–0.91***	***0.003***
Symptomatic	1.01	0.85–1.19	0.95			
*Ipsilateral brain infarcts*	***1.52***	***1.30–1.78***	** < *0.001***	***1.49***	***1.27–1.75***	***0.003***
Cardiovascular risk factors						
*Coronary artery disease*	***1.36***	***1.16–1.60***	** < *0.001***	1.14	0.97–1.34	0.12
*Hypertension*	***1.36***	***1.00–1.84***	***0.01***	1.17	0.86–1.59	0.32
Dyslipidemia	1.12	0.95–1.31	0.18			
*Chronic obstructive pulmonary disease*	***1.61***	***1.30–2.00***	** < *0.001***	***1.42***	***1.14–1.78***	***0.002***
*Severe chronic kidney disease*	***1.99***	***1.41–2.81***	** < *0.001***	***1.56***	***1.10–2.21***	***0.01***
Current smoking	1.01	0.86–1.18	0.95			
*Diabetes*	***1.27***	***1.08–1.49***	***0.003***	***1.21***	***1.03–1.42***	***0.02***
Peripheral artery disease, *n* (%)	0.93	0.70–1.23	0.69			
Aortic disease (aneurysm/dissection), *n* (%)	1.54	0.76–3.11	0.23			
Carotid score	0.97	0.91–1.03	0.26			
*Ipsilateral carotid plaque thickness (mm)*	***1.08***	***1.01–1.14***	***0.02***	1.04	0.97–1.11	0.26
Ipsilateral CW anatomy						
Multiple incompleteness (Type 0)	1.17	0.86–1.59	0.32			
*One part incomplete, other hypoplastic*	***1.32***	***1.09–1.60***	***0.005***	***1.24***	***1.01–1.51***	***0.04***
One part incomplete, other normal	1.14	0.95–1.36	0.17			
Both parts hypoplastic	0.86	0.66–1.13	0.29			
One part hypoplastic, other normal	0.87	0.73–1.04	0.13			
*Both parts normal*	***0.78***	***0.62–0.97***	***0.03***	0.85	0.68–1.07	0.17
Plaque type						
*Calcified*	***1.62***	***1.38–1.91***	** < *0.001***	***1.40***	***1.13–1.73***	***0.002***
*Fibrotic*	***0.75***	***0.58–0.95***	***0.02***	0.89	0.67–1.19	0.44
*Lipid-rich*	***0.68***	***0.58–0.81***	** < *0.001***	0.81	0.65–1.00	0.054
Exulcerated	1.10	0.92–1.32	0.31			

*CW* circle of Willis

Several cardiovascular comorbidities were significantly associated with mortality in univariable analyses, including coronary artery disease (HR 1.36, 95% CI 1.16–1.60; *p* < 0.001), hypertension (HR 1.36, 95% CI 1.00–1.84; *p* = 0.01), chronic obstructive pulmonary disease (HR 1.61, 95% CI 1.30–2.00; *p* < 0.001), severe chronic kidney disease (HR 1.99, 95% CI 1.41–2.81; *p* < 0.001), and diabetes (HR 1.27, 95% CI 1.08–1.49; *p* = 0.003). In multivariable models, chronic obstructive pulmonary disease (HR 1.42, 95% CI 1.14–1.78; *p* = 0.002), severe chronic kidney disease (HR 1.56, 95% CI 1.10–2.21; *p* = 0.01), and diabetes (HR 1.21, 95% CI 1.03–1.42; *p* = 0.02) remained independent predictors of mortality.

With regard to intracranial collateral anatomy, univariable analysis demonstrated that a CW configuration characterized by one part incomplete and the other hypoplastic (type 1) was associated with increased mortality (HR 1.32, 95% CI 1.09–1.60; *p* = 0.005), whereas a completely normal CW configuration predicted improved survival (HR 0.78, 95% CI 0.62–0.97; *p* = 0.03). Multivariable analysis confirmed type 1 CW anatomy as an independent determinant of mortality (HR 1.24, 95% CI 1.01–1.51; *p* = 0.04).

Plaque composition also showed significant associations with long-term mortality. In univariable analyses, calcified plaques were associated with increased mortality (HR 1.62, 95% CI 1.38–1.91; *p* < 0.001), whereas fibrotic (HR 0.75, 95% CI 0.58–0.95; *p* = 0.02) and lipid-rich plaques (HR 0.68, 95% CI 0.58–0.81; *p* < 0.001) were associated with improved survival. In multivariable analysis, calcified plaque morphology remained an independent predictor of mortality (HR 1.40, 95% CI 1.13–1.73; p = 0.002). Exulcerated plaques were not significantly associated with mortality.

## Discussion

The present study demonstrates that ipsilateral cerebral infarcts and long-term mortality in patients undergoing CEA are determined not by a single vascular feature, but by the convergence of carotid plaque vulnerability, global extracranial carotid disease burden, and intracranial collateral capacity. Specifically, ipsilateral infarcts were associated with more advanced bilateral carotid disease, increased plaque thickness, exulcerated plaque morphology, and severely compromised CW anatomy, while overall mortality was predicted by the presence of cerebral infarcts, compromised CW anatomy, and calcified plaques. Multivariable analyses revealed distinct associations between worse carotid scores and watershed infarcts, and between thicker, exulcerated plaques and territorial infarcts, suggesting divergent mechanisms of cerebral injury. Taken together, these findings indicate that cerebrovascular events in this population reflect integrated structural and functional consequences of progressive vascular ageing rather than isolated focal lesions.

### Why infarcts matter clinically

At the level of clinical manifestation, cerebral infarcts represent a critical integrator of cerebrovascular vulnerability. The strong association between ipsilateral cerebral infarcts and symptomatic carotid disease observed in our cohort is consistent with prior studies [4, 16, 17]. Beyond infarct presence, infarct size has emerged as an important determinant of perioperative risk and timing of intervention. Current guidelines recommend delaying revascularization in patients with infarcts exceeding one-third of the middle cerebral artery territory [7]. More refined volumetric analyses have shown that infarct volumes ≥ 4,000 mm^3^ independently predict 30-day postoperative stroke (OR 4.6; 95% CI 1.1–19.1; *p* = 0.03) [4, 5], supporting deferred intervention. Conversely, early revascularization within two weeks confers the greatest benefit in symptomatic patients with smaller infarcts [4, 5, 7], while silent infarcts in asymptomatic patients identify subgroups with increased long-term stroke risk [18] suggesting a potential role for preventive carotid revascularization [6, 7]. From a geroscience perspective, infarcts may therefore be viewed not only as acute events but also as markers of reduced cerebrovascular resilience in aging individuals.

#### Hemodynamic versus embolic mechanisms

Beyond infarct presence, understanding how infarcts arise requires consideration of underlying injury mechanisms. Lower carotid scores, reflecting higher-grade and combined ipsilateral and contralateral extracranial carotid artery stenoses, were associated with an increased frequency of cerebral infarcts, either through a direct haemodynamic effect resulting in critical cerebral hypoperfusion [19] or indirectly via the coexistence of multiple and potentially more emboligenic plaques [20, 21]. The pathophysiology of territorial and watershed infarcts remains complex and controversial, as reduced perfusion, embolic phenomena, or their combination may contribute to both infarct types. Watershed infarcts, in particular, may arise from systemic microemboli blocking distal cerebral arteries [22], followed by reduced perfusion in border-zone regions that limits embolus clearance and culminates in infarction [23].

In our study, lower carotid scores independently predicted watershed rather than territorial infarcts in multivariable analysis, supporting a predominantly haemodynamic mechanism. In addition to reduced inflow, effective cerebral perfusion critically depends on intact autoregulatory mechanisms that dynamically adjust arteriolar tone in response to changes in perfusion pressure [24]. Studies in older adults with carotid artery stenosis demonstrate that cerebral autoregulatory function and inter-hemispheric blood flow compensation are frequently impaired, reflecting dysfunction of resistance vessels and the cerebral microcirculation rather than stenosis severity alone. Aging-related endothelial dysfunction, reduced nitric oxide bioavailability, arterial stiffening and capillary rarefaction blunt these compensatory responses, thereby increasing susceptibility to watershed infarction under conditions of global hypoperfusion. In contrast, we found that exulcerated plaques were significant determinants of territorial infarcts, pointing toward distal embolization as the dominant mechanism. This interpretation is consistent with studies in embolic stroke of undetermined source [25–27] as well as investigations in confirmed cardioembolic stroke [28]. Aging likely amplifies both pathways by reducing vascular compliance, impairing endothelial function, and limiting compensatory reserve.

### Circle of Willis: intracranial collateral capacity

The ability of the brain to tolerate hemodynamic challenges is further shaped by intracranial collateral capacity. Incomplete CW anatomy has been associated with the presence of cerebral infarcts [12] as well as with unfavourable stroke outcomes [13]. In line with these observations, the current study demonstrated a significant correlation between cerebral infarcts and severely compromised CW configurations, defined by multiple incompleteness or a combination of incompleteness and hypoplasia, whereas a complete CW appeared protective. Normal CW anatomy is a prerequisite for effective collateral recruitment under conditions of reduced inflow from a stenosed ICA, as demonstrated by a 4D phase-contrast MRI study [29]. The reported association between incomplete CW anatomy and intraplaque haemorrhage further supports a shared aging-driven vascular vulnerability affecting both extracranial and intracranial compartments [30]. These findings suggest that aging-related impairment of vascular adaptability may link extracranial plaque vulnerability and intracranial collateral insufficiency.

### Plaque vulnerability phenotypes

At the plaque level, embolic risk is driven by qualitative features that reflect biological instability rather than stenosis severity alone. Exulcerated plaques were identified in 22.6% of our study population, consistent with previous CTA-based investigations [31]. Vulnerable carotid plaques have been extensively studied in carotid atherosclerosis and stroke [32, 33], with multiple reports demonstrating higher rates of cerebral ischemia in patients with high-risk plaque features across a wide range of stenosis severities, including low-grade stenosis using multimodal imaging [34], MRI-based studies [35], and high-grade stenoses assessed by conventional angiography [36]. A meta-analysis by Baradaran et al. demonstrated a significant association between plaque ulceration detected on CTA and cerebral infarcts in symptomatic patients (OR 2.2) [37]. Exulcerated plaques [38] and other high-risk non-stenosing carotid plaques [33] have also been shown to correlate strongly with embolic stroke of undetermined source. Such features likely reflect chronic inflammatory activation, which intensifies with advancing age.

### Plaque thickness as vulnerability marker

Quantitative plaque burden further refines assessment of vulnerability. Maximum plaque thickness has been proposed as a quantitative marker of plaque vulnerability and has been associated with total plaque volume, future major cardiovascular events, and stroke occurrence in asymptomatic patients [39, 40]. Significant correlations between plaque thickness ≥ 3 mm and ipsilateral brain infarcts of undetermined source have been reported [33, 38, 41, 42]. Zhao et al. demonstrated a strong relationship between maximal wall thickness and other high-risk plaque features, including lipid-rich necrotic core, intraplaque hemorrhage, and surface disruption, in a large cohort of symptomatic patients, suggesting that plaque thickness may be a stronger discriminator of plaque vulnerability than stenosis degree [43]. In the context of aging, increasing plaque thickness may therefore serve as an imaging surrogate of cumulative vascular injury.

### Mortality and aging signals

Ultimately, the long-term impact of cerebrovascular disease is reflected in survival. The significant association between overall mortality and the presence of cerebral infarcts observed in our study is consistent with previous reports demonstrating higher long-term mortality in CEA patients with silent brain infarcts [6]. To our knowledge, the relationship between survival and CW anatomy has rarely been investigated in carotid atherosclerosis. One study examining 1.5-year survival after carotid revascularization reported no significant difference between incomplete and complete CW configurations [44], whereas our findings indicate that compromised CW anatomy is associated with long-term mortality. We also observed significantly higher mortality in men, in line with prior reports of sex-related differences in plaque burden and vulnerability [45].

Notably, vascular calcification emerged as a strong mortality signal. Calcified plaques, often regarded as markers of plaque stability, were independently associated with increased mortality in our cohort, whereas lipid-rich and exulcerated plaques were not. This finding aligns with observations from the Rotterdam Study, where larger calcification volumes were associated with a higher prevalence of intraplaque haemorrhage [46], suggesting that calcification may reflect advanced systemic atherosclerosis rather than plaque stability per se. From a geroscience perspective, these imaging phenotypes—plaque vulnerability, bilateral extracranial disease burden, impaired intracranial collateral capacity, and vascular calcification—may represent structural and functional manifestations of advanced vascular aging.

Taken together, these findings highlight the importance of viewing cerebrovascular imaging through the lens of aging biology. Plaque vulnerability, bilateral extracranial disease burden, impaired intracranial collateral capacity, and vascular calcification appear to cluster as manifestations of advanced vascular aging, collectively increasing susceptibility to cerebral ischemia and adverse long-term outcomes. Rather than representing isolated pathological features, the co-occurrence of plaque vulnerability, bilateral carotid disease, impaired autoregulatory reserve, compromised collateral anatomy, and vascular calcification is consistent with a systemic process of unhealthy vascular aging that simultaneously affects large extracranial arteries, intracranial collaterals, and the cerebral microcirculation [47, 48]. In addition, although extracerebral vascular disease manifestations such as peripheral artery disease and aortic pathology were common in our cohort, they did not show independent associations with cerebrovascular or mortality outcomes, suggesting that carotid imaging phenotypes may capture aspects of systemic vascular aging not fully reflected by conventional comorbidity measures.

### Limitations

This study has several limitations. CT is less sensitive in detecting small recent or chronic infarcts, which may result in underestimation of infarct burden. Only the presence and type of infarcts were evaluated; infarct volume was not assessed, and volume-based analyses stratified by symptomatic status were not performed. Plaque thickness was used instead of volumetric plaque assessment, and qualitative plaque characterization was applied rather than automated segmentation, which may underestimate vulnerable plaque components due to overlapping attenuation values. Photon counting CT technology could have improved plaque component differentiation. As a retrospective cohort study, our analysis is subject to potential treatment indication bias and variability in imaging timing relative to symptom onset; however, adjustment for symptomatic status likely mitigates these effects. Cause-specific mortality data were unavailable, necessitating reliance on overall mortality. These limitations reflect real-world clinical constraints but do not diminish the robustness of the observed associations.

## Conclusion

In this large, real-world cohort of patients undergoing carotid endarterectomy, ipsilateral cerebral infarcts and long-term mortality were associated with a constellation of imaging phenotypes reflecting plaque vulnerability, bilateral extracranial carotid disease burden, impaired intracranial collateral capacity, and vascular calcification. The differential associations of watershed and territorial infarcts with hemodynamic compromise and embolic plaque features, respectively, underscore the coexistence of distinct but interacting mechanisms of cerebrovascular injury.

From a geroscience perspective, these findings support the concept that carotid artery disease and its cerebral consequences are manifestations of unhealthy vascular ageing, a systemic process simultaneously affecting large extracranial arteries, intracranial collaterals, and the cerebral microcirculation. Impaired autoregulatory reserve, reduced vascular adaptability, and cumulative structural remodelling appear to limit cerebrovascular resilience in ageing individuals, predisposing to ischaemic brain injury and adverse long-term outcomes. Comprehensive cerebrovascular imaging may therefore serve not only as an anatomical assessment tool, but also as a surrogate marker of biological vascular age, with important implications for risk stratification and individualized management of carotid artery disease.

## Data Availability

The datasets generated and analyzed during the present study are not publicly available due to privacy considerations. However, anonymized data may be obtained from the corresponding author upon reasonable request and will be provided in compliance with institutional and ethical regulations.

## Abbreviations

CEA: Carotid endarterectomy
CI: Confidence interval
CTA: CT angiography
CW: Circle of Willis
HR: Hazard ratio
ICA: Internal carotid artery
NASCET: North American Symptomatic Carotid Endarterectomy Trial
OR: Odds ratio
SD: Standard deviation
